# Supplementary figures and images for: A simple pyrocosm for studying soil microbial response to fire reveals a rapid, massive response by Pyronema species
Source: PLoS One. 2020 Mar 4;15(3):e0222691. doi: 10.1371/journal.pone.0222691 (PMC7055920; doi:10.1371/journal.pone.0222691)

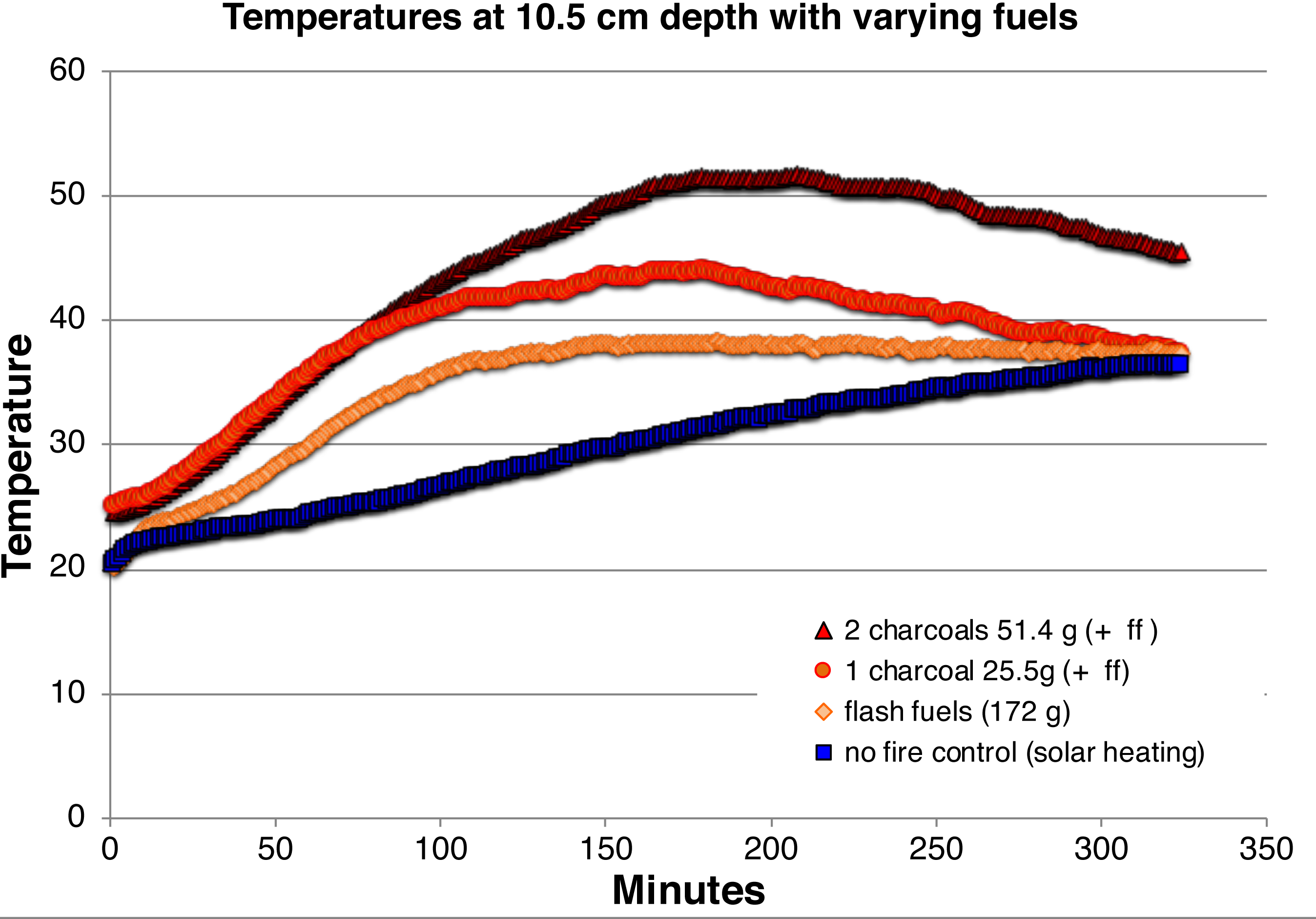

Supplement: S1 Fig — 172 gm of flash fuels caused temperatures to rise slightly at 10.5 cm below the surface, but ultimately achieved the same peak temperature as solar heating in an unburned pyrocosm monitored simultaneously. Pyrocosms with the same flash fuel load, but one or two charcoal briquettes (25–51 g) heated more rapidly and achieve higher peak temperatures. (TIF) [file pone.0222691.s001.tif]

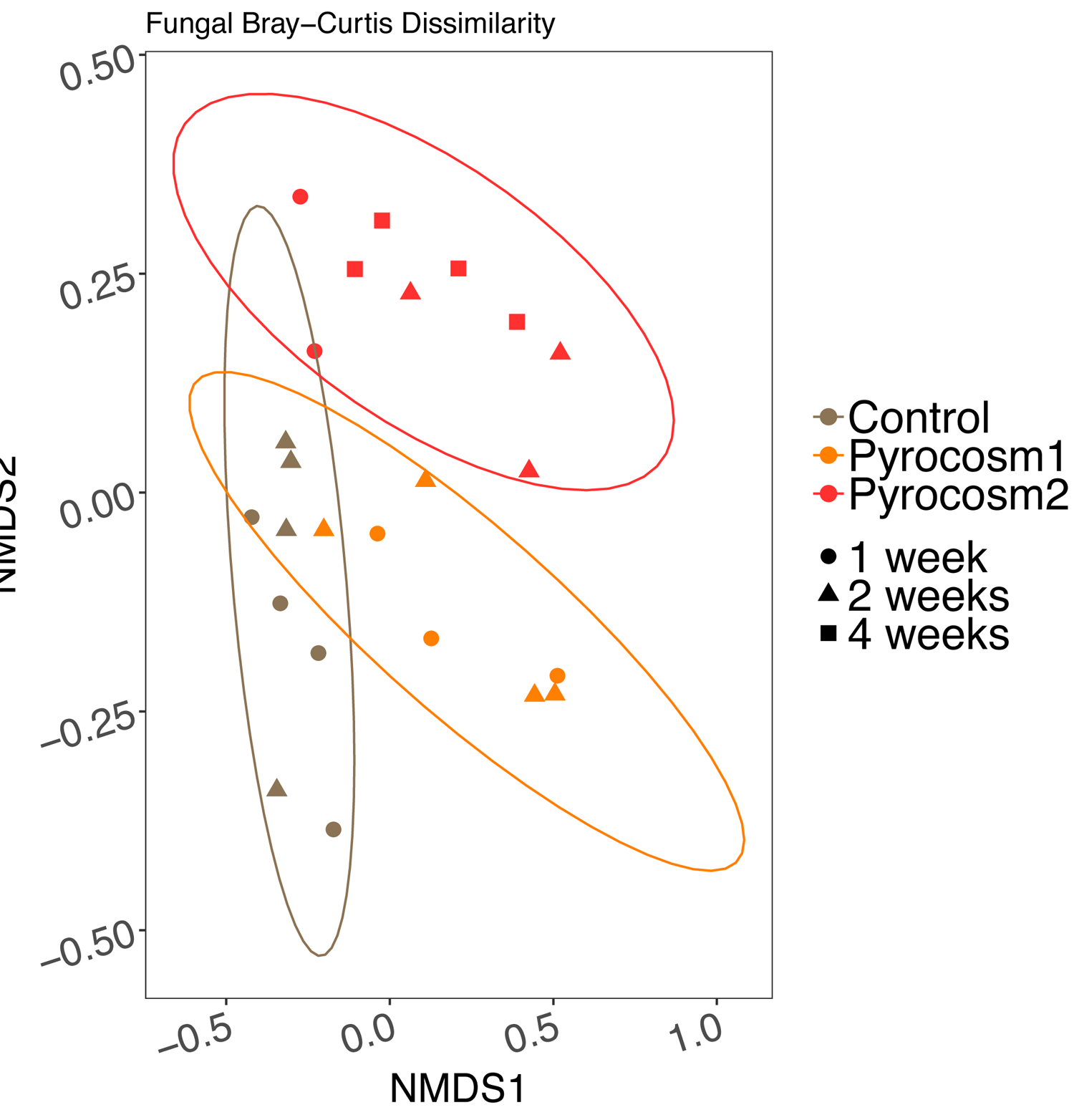

Supplement: S2 Fig — Samples were rarified to 3950 reads/sample. Adonis R2 = 0.33 and 0.19 for Bray-Curtis (A) or Jaccard (B) respectively. (TIF) [file pone.0222691.s002.tif]

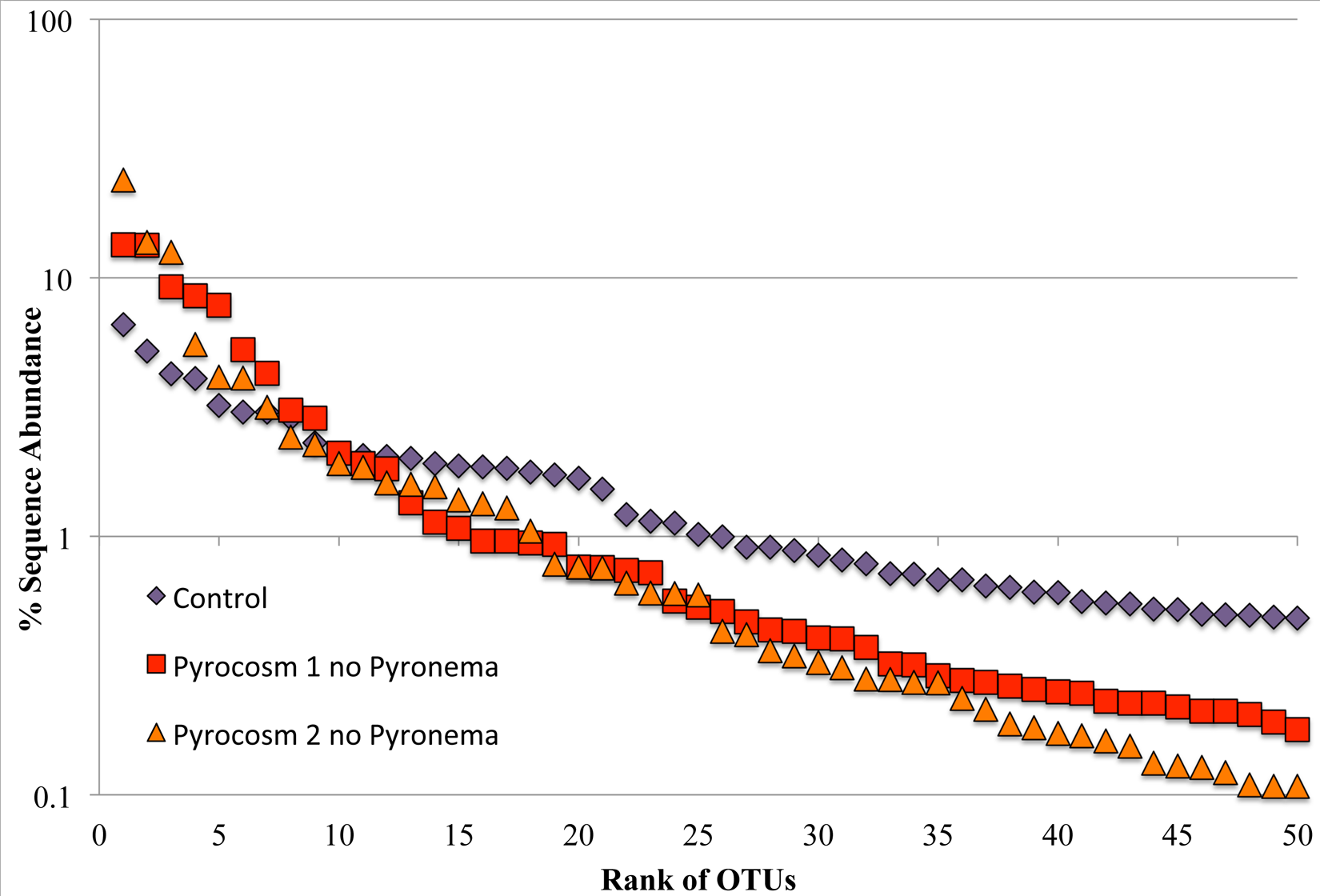

Supplement: S3 Fig — Top 50 most abundant OTUs are shown. (TIF) [file pone.0222691.s003.tif]
