# Supplementary material for: A simple pyrocosm for studying soil microbial response to fire reveals a rapid, massive response by Pyronema species
Source: PLoS One. 2020 Mar 4;15(3):e0222691. doi: 10.1371/journal.pone.0222691 (PMC7055920; doi:10.1371/journal.pone.0222691)
Supplement: S1 File — (DOCX) [file pone.0222691.s004.docx]

**File S1 Notes on assembly and use of pyrocosms.**

**Precise placement of thermocouples**

Because the peak temperature is a function of the log of depth, the precise vertical placement of the thermocouples is critical to obtain an accurate estimate of peak temperatures. To achieve this we take a thin metal rod (photo below), marked with the depths at which we plan to install thermocouples, and stand it in the bottom of the bucket. Test soil or sand is then filled in until it is level with the first marked depth. A thermocouple is inserted from a drill hole in the side of the bucket. The inexpensive, thin, wire, k-type thermocouples are ideal for this, because they can be bent slightly near the bucket edge so that the tip presses down on the level soil surface; we obtained ours from Amazon.com. The wire can then be taped in place on the outside of the bucket with high temperature aluminum tape (Scotch Brand 425 5130-06-05). Additional test soil or sand is then added on top of the thermocouple wire, until the next depth mark is reached, and then the another thermocouple wire is installed in the same manner. When all desired thermocouples are placed, the metal rod is carefully pulled out.


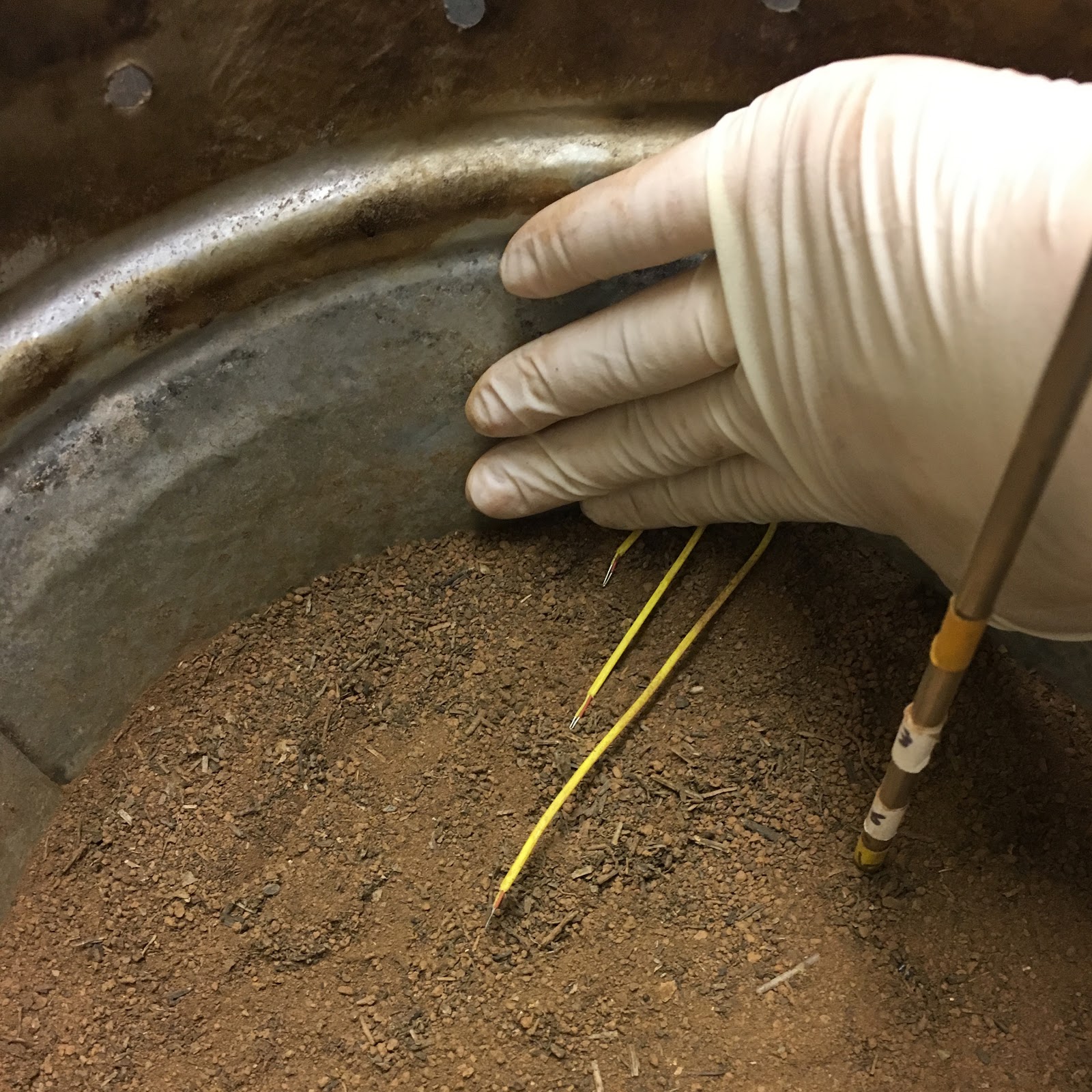


Only two thermocouples/unit are needed to obtain accurate estimates of the temperature profiles in the center of the units because the relationship between peak temperature and the log of depth is quite strong (Fig 2B). If the inexpensive wire thermocouples are used, we recommend placing them at least 6 cm from the surface so that they do not experience temperatures above their limit (200 C), but the safe depth will vary with the fuel load used. We also recommend placing them at least 2 cm from the bottom, because there is a slight edge effect of the bucket even in the vertical direction. Label the plug ends of the thermocouples so that the position of the thermocouples can be easily known even when the bucket is buried.

**Placing the pyrocosms in the soil**

To avoid movement of the thermocouples minimized shaking of the assembled pyrocosm when moving it from the lab to the burn site. Dig a hole large enough for the pyrocosm to fit in, such that the soil levels inside and outside the pyrocosm are aligned. Dig a small trench on the side of the unit where the thermocouples are inserted, and run the thermocouple wires through this trench before filling it in with soil (see yellow wires emerging from the soil in Fig 1D). Clear any flammable material away from the area around the pyrocosm.

**Fuel and ignition options**

Starting a larger charcoal fire and removing the burning coals from the pyrocosm when a selected temperature is reached at a target depth is the way we initially used the pyrocosms. Although this works, it is less convenient and less accurate than using a selected fuel load and letting it burnout completely. Excess charcoal is inconvenient because it requires live monitoring of the unit for a couple hours or more, and it is inaccurate because the soil temperatures keep rising after the coals are removed.


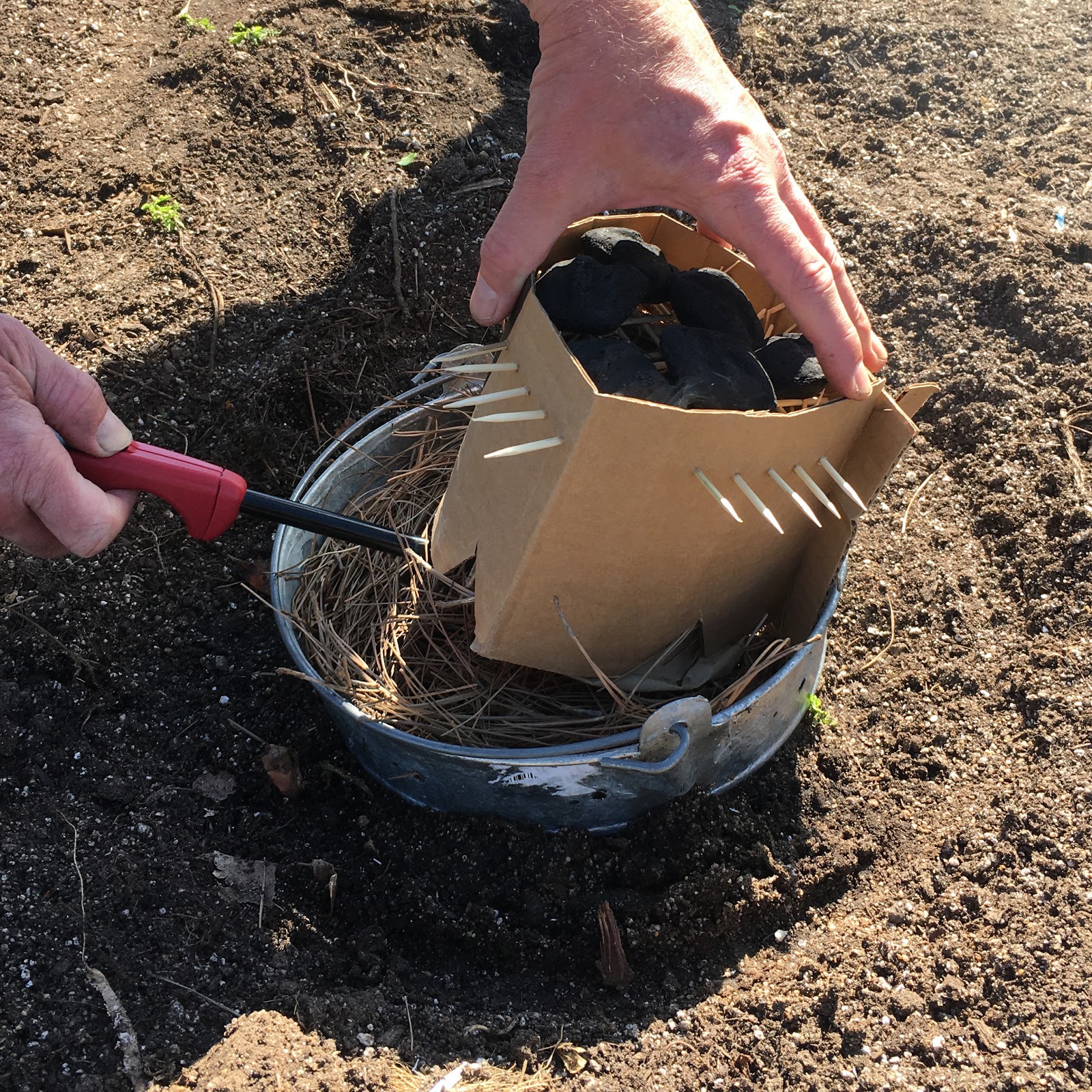

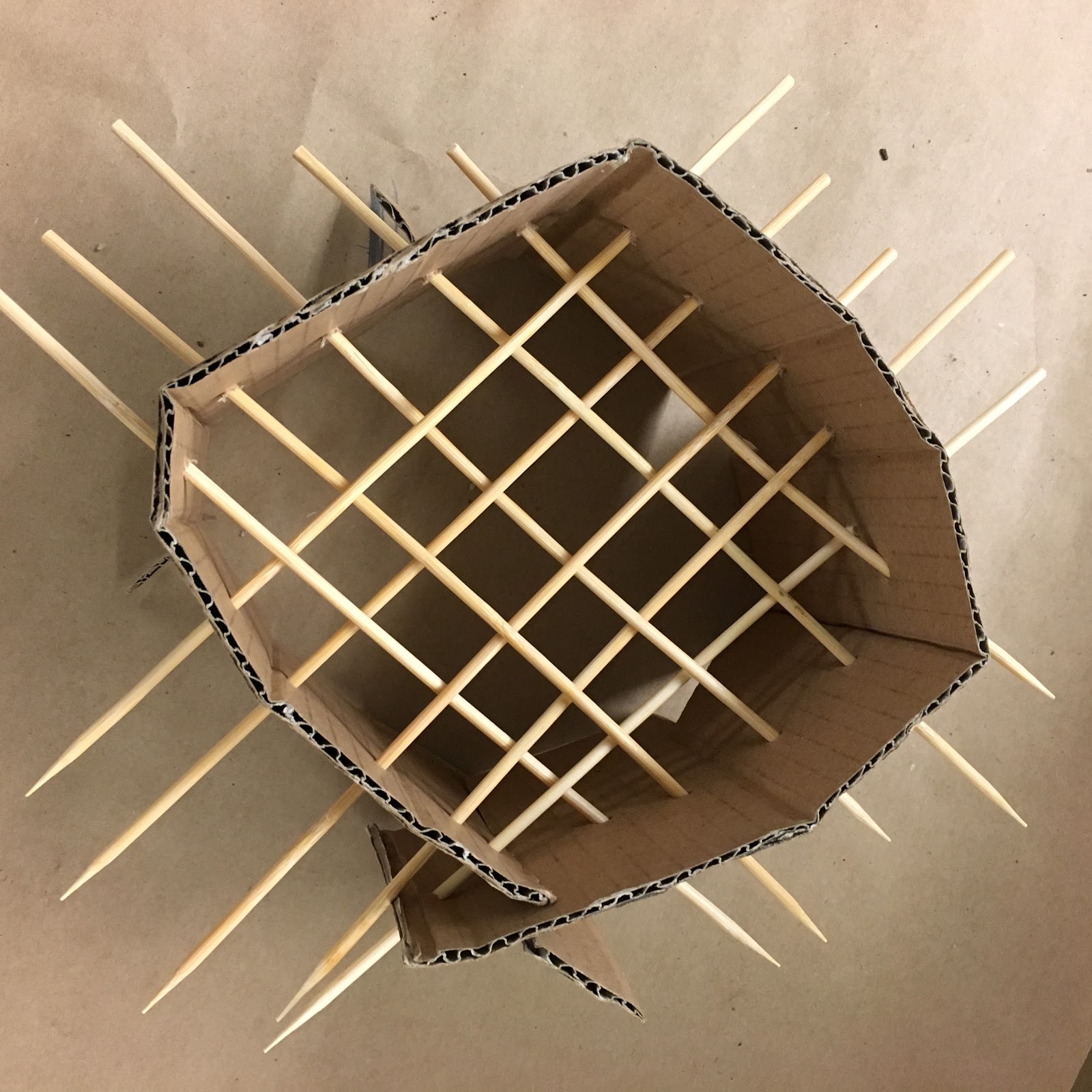
Efficient ignition of the charcoal is the goal of the cardboard cone shown below and in Fig 1b. The cardboard ring is usually 30 to 38 g and stands about 12 to 15 cm tall. The cut-out triangles at the base are needed for aeration. A small platform is constructed about 4 cm from the top with 10 wooded skewers.

A single sheet of newspaper is crumpled up below this, 10 g of wooden toothpicks are added on top, and the charcoal is added last. Light it from below, and tend it for a few minutes with a metal tool to prevent it from falling over prematurely while it is burning. This simple construct usually works well if it is not overfilled.

Alternatively “match light” charcoal can be placed directly on the litter with no elaborate cone or additional fuel. This works very well, but we stopped using this approach because we are now measuring post-fire soil chemistry and wanted to avoid the addition of petroleum products that might be detected later.

**Temperature equilibration**

To achieve reproducibility in temperatures the starting temperature of the soil needs to be controlled or at least considered when setting up an experiment. Once the units have been buried in the soil for multiple hours they become buffered by the ambient soil temperature, and all units will have nearly identical starting temperatures if the experiments are started at the same time of day. However, there is some diurnal heating and cooling of the soil, and this is accentuated if they are exposed to solar heating. Thus starting one experiment in the morning and a different one in late afternoon will result in slight differences in peak temperatures that are unrelated to fuel load. If the units are left indoors overnight, and buried in the soil just prior to use, then the starting temperatures can be controlled independent of time of day.

**Watering to initiate *in situ* incubation**

The addition of water is likely to be necessary to initiate microbial recolonization because these soils are quite dry. Dry soils are also difficult to core because they are not retained in the corer. We add water in the form of crushed ice, because it melts slowly and enters the soil fairly evenly. We have targeted moisture contents of 20 to 30% by using pre-burn moisture contents and calculating the additional water needed. However the burn dries the soil further, making these estimates imprecise, and we have not experimented with optimizing the water content. Once watered, we cover the top of the bucket with foil or a metal pan to lessen evaporation and prevent solar heating and additional precipitation. We do not usually put drain holes in the bottom of the buckets, but if they are being incubated in the open during seasons where rain is likely then drain holes may be necessary.

**Using pyrocosms as a source for post-fire soils**


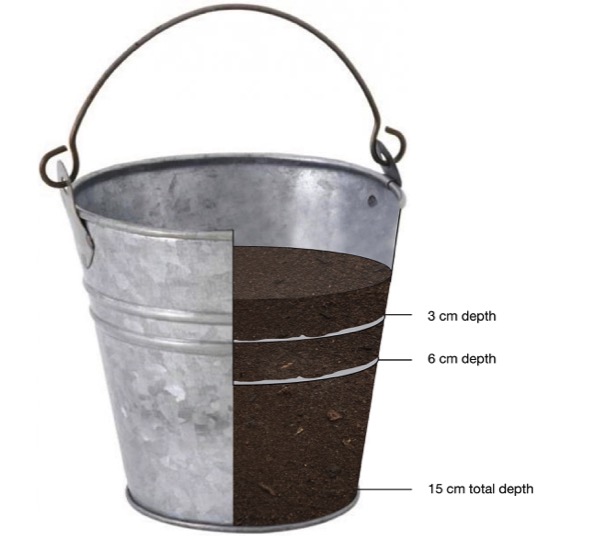

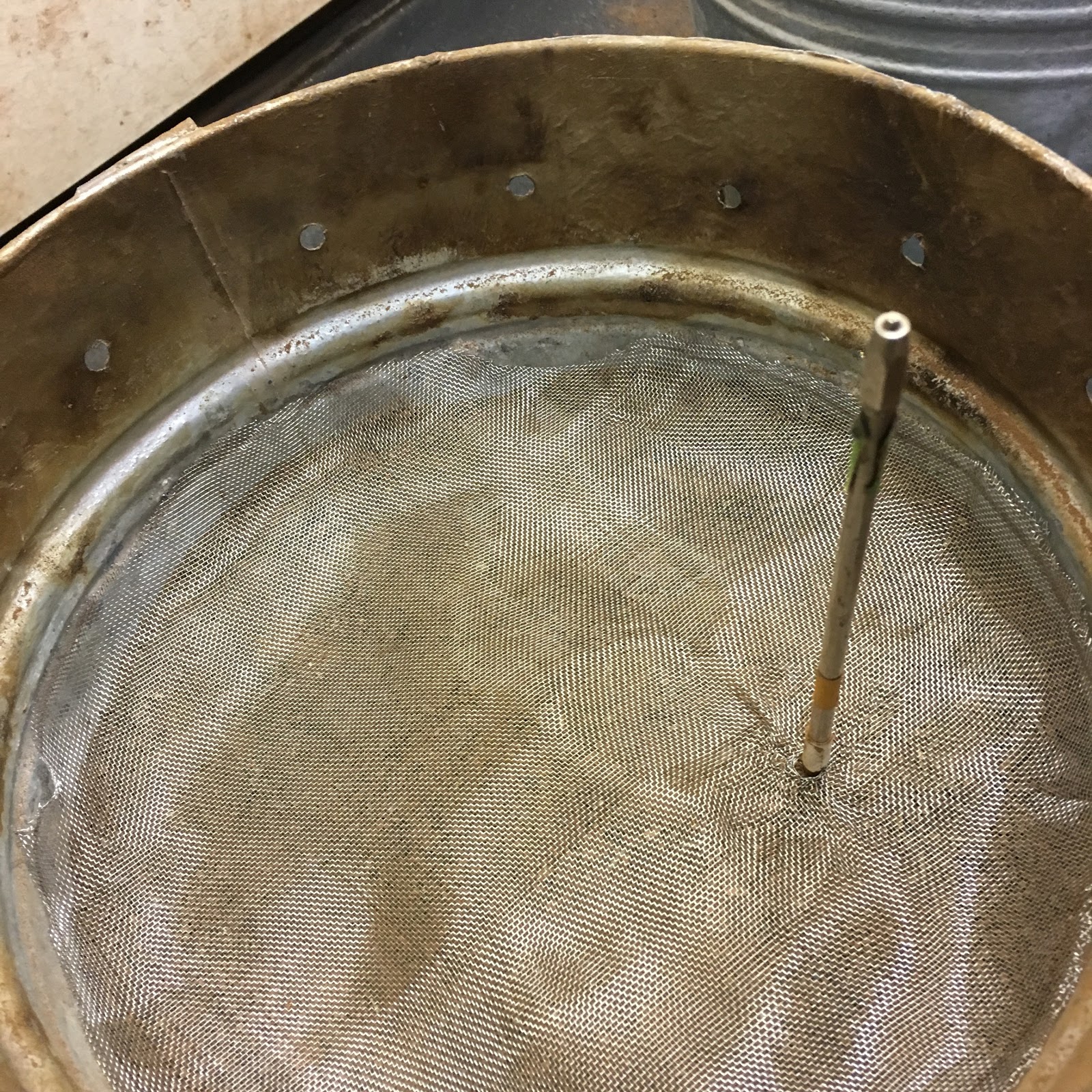
Temperature-defined zones of post-fire soils can be removed and used to incubate organisms of interest *in vitro*. The most efficient way to do this is to determine the depth of this zone in advance (via fuel manipulation), and then place stainless steel screens (~1mm hole size) below and above the zone of interest when assembling the pyrocosm (inserted photo above). This makes it easier to cleanly remove a zone, and also evens out the temperature variations within the zone. If test soil is limited one can fill most of the lower pyrocosm volume with sand, and then layer the test soil on top of it staring at the depth required. Thermocouples can be placed directly in the targeted zone to obtain an accurate measure of the peak temperatures reached. From our limited experiments with this approach we have found that soils that achieved high temperatures can be used without further sterilization to grow fast-growing pyrophilous fungus. It can also be frozen at -80 C until one is ready to use it.
