# Supplementary material for: A simple pyrocosm for studying soil microbial response to fire reveals a rapid, massive response by Pyronema species
Source: PLoS One. 2020 Mar 4;15(3):e0222691. doi: 10.1371/journal.pone.0222691 (PMC7055920; doi:10.1371/journal.pone.0222691)
Supplement: Attachment — Submitted filename: Response to Reviewers.doc [file pone.0222691.s005.doc]

**General comments about the revision:** We thank the reviewers for their careful reading of the manuscript and their many useful edits, suggestions and comments. They have certainly helped us refine our message and the presentation of results in this revision.

Comments on content are addressed below in the line-by-line list by reviewer. Small edits (spelling, grammar, word use changes) suggested have been made and can be found in the marked up version of the revision but will not be covered below.

The most general criticism made was about the limited replication (two experiments) in the biological part of the study. We concede that this limits the organismal inferences that can be drawn, and we had acknowledged this in various parts of the first draft. However, it serves the purpose of demonstrating that our simple experimental system works to stimulate at some pyrophilous fungi, and reviewer 2 and 3 recognized this, but thought it should be clarified. Similarly at least two reviewers commented our description/presentation of the “Goldilocks zone”, and though found it interesting, thought that it was not well incorporated or was overly discussed.

In response to these comments we clarified our goals and role of the biological experiments in the introduction. We did this by outlining our thermo-chemical gradient model in the third and fourth paragraphs. This succinctly summarizes the way we theorize that heat, soil depth and altered soil chemistry interact to create a predictable, depth-structured habitat for microbes in post-fire soils. In the last paragraph of introduction, we make it clear that: 1) our primary goal was to develop an experimental system that allows the post-fire soil system to be manipulated at a fine scale; 2) the forest soil experiments were essentially a pilot study to show that our experimental system has biological relevance, and 3) the heating results are used to refine our concepts of the Goldilocks and necromass zones in our model.

Below we address the specific concerns of the three reviewers.

Reviewer 1

Thanks for your comments and edits. However, we are not completely sure that we understand exactly what your concern about the lack of replication is referring to. If it is the biological replication, we concede that two reps and one control is not enough to generalize, but as discussed above we have now clarified that they serve primarily as a proof of concept. If concern is referring to the heating experiments then this is incorrect. As we showed with regressions that soil heating is highly predicable from our data. Specifically peak temperatures at difference depths are related by the ln of the depth (Fig2b), and peak soil temperature at a selected depth are a function of the mass of fuel (Fig 2C). The R2 on all these regressions is quite high, and replication of specific fuel loads is not necessary to demonstrate this. Nevertheless, we show that replicated fuel loads result in nearly identical heating profiles (Fig 2A). This can also be seen in Fig 2C for fuel load just under 300g.

In terms of “showing readers how to alter fuel loads” we have made the methods clearer now, but we think that part of the problem may be that the reviewer apparently missed a supplementary file (S1) that goes into more detail on construction and uses of the pyrocosms.

We have now reworked the introduction to better justify the development of the pyrocosms. This is specifically addressed in the last paragraph of the introduction. Thank you for this suggestion.

Former line 22 - Yes, we think “massive” is an apt descriptor for a response that increases the sequence abundance of an organism from barely detectable levels to roughly 60% of all sequence. This is an order of magnitude more dominant than is typical of soil fungi in sequence studies.

Former line 25 - The reason reduction in richness can occur without large changes to the other fungal taxa is because we use a relative abundance measure (i.e., sequence %). Thus large increases in a very small subset of taxa (e.g. *Pyronema*) decreases the apparent abundance of other taxa. The taxa “lost” were those that had very small abundance in the pretreatment condition, but taxa that were reasonably abundant are retained at similar levels. This is covered more in the results and discussion.

Former line 29 - now reworded to make the antecedent of “it” clear.

“Pyrolyzed” is now qualified were it needs to be and defined more carefully. The reviewer’s comment about the need for low oxygen for pyrolysis may be technically correct, but in practice this is clearly not a limitation in soil systems, or even soil surfaces, where charred remains are common.

Former line 33 - Pyrophilous is now used uniformly

Former line 115 - We have revised the description of the pyrocosm setup to make it clearer and corrected the volume discrepancies that other reviewers caught as well. We also point to the supplemental notes (File S1) that have addition images and details.

Former line 158 and elsewhere the number and type of pyrocosm experiments is now clarified.

The selection of depths for the thermocouples was somewhat arbitrary but is explained on lines 310-313: “These depths were selected to insure that the wire thermocouples did not experience temperatures above 200ºC across the range of fuel loads tested.”

Former line 322; Method of drying litter is now explained

Former line 322 - F-layer and litter layer density was not measured; the tamping down was meant to approximate it, but we have no measure of how well we achieved that. However, it was completely incinerated, and we show that such flash fuels causes negligible soils heating, so we doubt it matters for the reproducibility or realism of the experiments.

Former line 180-185 The use of charcoal is now explained more fully. Essentially we use charcoal because they burn very predictably, transfer heat well to the soil, are probably not too different from natural substrates in forest fires, and are readily available.

Former line 275 - The finding that peak temperatures at depth are achieved hours after the fire goes out and linger for ~40 minutes is absolutely not artefactual; it is caused by the large heat capacity of soil. This makes soil resistant to temperature change, but allows it to absorb and hold heat. Thus the heat source for soil at depth is the soil above it, not the active combustion. The result occurred in all the sand pyrocosms and both the soil pyrocosms, and it is likely to be greater in nature where the volume of soil is larger and there are not unheated edges. You can see evidence of this same effect in Smith et al paper cited where they burned log piles in nature.

**Reviewer 2**

The first comments about the value of this work being in the system, and the value of the sequencing being in the demonstration of its biological value is exactly what we had hoped to communicate. After reading the reviews and realizing that this was not obvious to everyone, we reworked the introduction as discussed above. Thank you for this clear insight.

We agree that our limited biological replication limits inference. Our attempt to point out these limitations and to extract those results that transcend them was part of the reason the discussion was longer than the reviewer desired. We have now dropped at least two paragraphs of results and discussion about the biological response. What we retained was focused on Pyronema, and explaining why the mycorrhizal “response” indicates the noise level.

The term “microbe” is not a taxonomic term - it’s simply a common name for small organisms. So yes, we see microfungi as a subset of microbes. Using this term particularly in the introduction is useful, because bacteria, and fungi both have to deal with the same fire and post-fire conditions and substrates, and they would both be assessed through very similar high-throughput sequence approaches. However, we have now tried to clarify exactly when we are referring to only fungi, which is obviously most of the time.

We retained the modeling of the Goldilocks and necromass zones but as discussed above have now incorporated better into the focus of the paper as part of a broader thermo-chemical model.

**Minor issues**

Former line 11 - Yes, we had given the wrong size for the buckets - nice catch.

Former line128 - flash fuel is now defined more clearly.

Former line 135 - the soil separation is now spelled out better.

Comment on former line 196 - if you check the results (Fig S1) heat from one briquette was easily detectable, and had a greater effect than a much larger mass of flash fuels.

Former line 213 - the depth of sampling is now specified (all the way to the bottom of the bucket)

Former line 227 - natural log is now specified but the truth is that log10 is almost as good.

Comment on former lines 301-303: - I think there is confusion here on what is being regressed and we have now corrected that wording to clarify it. Basically there are there two main regressions used in this study. 1) temperature regression, which uses measured temperatures at 2 depths to predict temperatures at unrecorded depths (i.e, Fig 2b); and 2) course fuel related regression - where grams of charcoal are used to predict peak temperatures at particular depths (Fig 2C). We agree completely with the reviewer that flash fuels cannot be predicted by the second regression, but that is not what we did. Instead we are just using the measured peak temperatures achieved by the flash fuels at two depths to predict temperatures at other depths. This is valid because it is simply a function of the heat capacity of the soil - any heat source can be modeled this way.

Former Line 20 page 21 - Other contamination sources are always possible with PCR, and that is why no-DNA and mock community controls are so important. What we show is that the contamination level is quite low. However, it is very common for mock communities or other control results to be contaminated by abundant sequences from other samples. This is because of “index switching” where the primer tags from one sample are recombined with the amplicon from another sample during PCR is common (see the Carlson et al 2012 paper cited). The more abundant the amplicon is, the more likely that it will experience index switching and end up looking like it was in samples it did not actually occur in. Thus, we think this is the most likely source of contamination. To avoid this, recent libraries have used double indexing (tags on both primers).

Former lines 33-39 p 22. The nominal mycorrhizal “stimulation” was clearly artefactual, and that is (and was) mentioned in the discussion. We think that from the collective knowledge of Russula species we can say with some confidence that it did grow in a soil environment without a host. It is likely that the few percent increase seen in the two burned pyrocosms relative to the one control is due to soil heterogeneity/sampling issues that are compounded by low replication. We think the level of “mycorrhizal response" is a nice benchmark, in that we can’t infer anything about taxa that were apparently stimulated to similar or lower levels.

Former line 69, page 23; 3950 was correct, not 6000. We corrected that now - nice catch!

Line 121-122 larger, glowing, heat sources certainly heat the soil more and result in higher temperatures at depth, but heat transfer follows the same physics even when the heating is less - that’s what our first regression shows. Even the slight heating from flash fuels showed the slow rise and the lingering effect (Fig S1); the main difference is that the peak temperature achieved is much lower/g of fuel. However, whatever peak temperature is achieved is will linger near that temperature for some time, and organisms need to endure it; that’s the point.

**Former** **Lines 109 etc, about ice:** Water needs to be added. This is dry soil, so without it little activity would be expected. The various amounts and ways it could be added are clearly another variable that could be tested. The advantage of ice is that it melts slowly and wets the soil thoroughly. We were worried liquid water would channel through the hydrophobic upper layers and create uneven wetting, and would need to be added a little at time to avoid this problem. With a single pyrocosm that could be done, but with multiple pyrocosms the ice is much easier and provides a very uniformly timed wetting. Is a good mimic of snow? It’s clearly not perfect, but it’s probably good enough. The one big Sierra fire (Rim Fire 2013) we followed was initially wetted by a snow event that melted quickly at the elevation that these soils came from, and it spawned fungal activity similar to that we observed in the pyrocosms.

Former line 176 - we have incorporated the necromass zone discuss into the paper better now (see bit on introduction), and reordered it in the discussion

**Former line202 p 29.** We had missed the Reazin et al. paper before, but have now incorporated it. It’s an excellent addition as it shows a massive *Pyromena* response in the field. We think this is the only such published example, although we have unpublished results from the Rim fire that show this too.

All sequence samples now have accession numbers rather than the XXXXX seen in the original submission

**Reviewer 3**

As discussed above we have now focused the paper on the pyrocosm system and the thermal-chemical gradient model, and this was partially in response to reviewers’ clear summary of the manuscript - we thank you for that. We have also clarified how many pyrocosms were involved and addressed the replication issues.

Thank as well for the many edits suggested - these have been incorporated, but they will not be addressed line-by-line here.

Former lines 61-62. Knowing that most pyrophilous fungi are saprobes really only scratches the surface in addressing the question of what they do in this chemically altered environment. We have now clarified our point by stating: “…, nor is it known what these fungi degrade and live on in the chemically altered post-fire environment.”

Former line 64-71 Discussion of ectomycorrhizal response to fire is now removed as suggested.

Former line 100: The distinction between wildfires and prescribed fires is now clarified throughout.

Former line 105 - The Goldilocks zone is now incorporated better.

Former line 138 - the postfire soil added was not depth stratified because it was collected from a disturbed pile of soil in the fire zone. This is now mentioned.

Former line 139 - the origins, use, and treatment of the litter and F-layer is clarified now.

Former line 149 - As explained in the set-up they were reused by removing ash following one experiment before adding fuel for the next. That is why we said “14 experiments” rather than 14 pyrocoms. We have clarified this now.

Former line 154 - Soil pyrocosms were also used for temperature experiments to the extent that the Ln relationship between depth and peak temperature are shown with soil (Fig 2B). This is indicated in the legend.

Former lines 206 - The amounts of water differed because we had not standardized the amount of water yet. As explained above the biological side of experiment was a pilot study. If it had not succeeded in producing a biologically relevant result, the rest of the study would not have gone forward. So essentially we were trying different water and soil to see if anything worked.

Former line 192-196 The soil experiments were actually run prior to the sand experiments. We only learned later that could create the same temperatures profiles with less manipulation by simply letting fewer charcoals burn completely. In the more detailed instructions on assembling pyrocosm (File S1) the difference between the two burning methods is discussed more, but the bottom line is that either works.

Former line 203 - the ice is now explained more fully, and the explanation for the differences in ice are explained above.

Former lines 213-217 - The coring number and depth is now clarified.

Former lines 221 - our choice of ITS 1 was basically so we could compare it to our own earlier data. - This is now explained.

Line 258 - Operational Taxonomic Unit had already been spelled out at first use of the OTU on line 228 (now line 376)

Line 273-315,and line 182. Temperature results are primarily from the sand pyrocosm, and the specifics are now indicated in the legend to Fig 2

Former lines 274-294 - The whole-genome sequencing section is germane to the system as a whole, because it makes it more experimentally more powerful. We spent a short paragraph on it, and we feel this is important so that others that might want to use the system are aware of the resources available.

Sincerely

Tom Bruns
